# COVID‐19 and sarcopenia-related traits: a bidirectional Mendelian randomization study

**DOI:** 10.3389/fendo.2023.1162936

**Published:** 2023-05-10

**Authors:** Chao Liu, Ningyuan Liu, Yi Zeng, Bo Xiao, Pingxiao Wang, Chuqiao Zhou, Yu Xia, Ziyue Zhao, Tao Xiao, Hui Li

**Affiliations:** ^1^ Department of Orthopedics, the Second Xiangya Hospital of Central South University, Changsha, China; ^2^ Orthopedic Biomedical Materials Engineering Laboratory of Hunan Province, Changsha, China

**Keywords:** COVID-19, sarcopenia, mendelian randomization, long COVID-19, aging

## Abstract

**Background:**

Emerging evidence suggested that coronavirus disease 2019 (COVID-19) patients were more prone to acute skeletal muscle loss and suffer sequelae, including weakness, arthromyalgia, depression and anxiety. Meanwhile, it was observed that sarcopenia (SP) was associated with susceptibility, hospitalization and severity of COVID-19. However, it is not known whether there is causal relationship between COVID‐19 and SP-related traits. Mendelian randomization (MR) was a valid method for inferring causality.

**Methods:**

Data was extracted from the COVID‐19 Host Genetic Initiative and the UK Biobank without sample overlapping. The MR analysis was performed with inverse variance weighted, weighted median, MR-Egger, RAPS and CAUSE, MR-APSS. Sensitivity analysis was conducted with MR-Egger intercept test, Cochran’s Q test, MR-PRESSO to eliminate pleiotropy.

**Results:**

There was insufficient result in the MR-APSS method to support a direct causal relationship after the Bonferroni correction. Most other MR results were also nominally consistent with the MR-APSS result.

**Conclusions:**

Our study first explored the causal relationship between COVID-19 and SP-related traits, but the result indicated that they may indirectly interact with each other. We highlighted that older people had better absorb enough nutrition and strengthen exercise to directly cope with SP during the COVID-19 pandemic.

## Introduction

The coronavirus disease 2019 (COVID-19) has evolved into an ongoing global pandemic affecting more than 600 million people, resulting in nearly 7 million deaths (1). Age, concurrent frailty and comorbidities were associated with higher risk of being positive for COVID-19, hospitalized and mortality (2). Meanwhile, accumulating evidence suggests that COVID-19 survivors could experience various sequelae, mainly including weakness, arthromyalgia, depression, anxiety and memory loss (3). Due to the complicated pathogenesis of COVID-19, the extremely challenging pandemic has forced people to live with COVID-19, which means it is important to evaluate the relevance between COVID-19 and other comorbidities.

Sarcopenia (SP) is the loss of skeletal muscle mass associated with aging which causes an involution of muscle strength, and/or low physical performance (4). Skeletal muscle-related traits have been widely reported in both acute COVID-19 and post-acute sequelae of COVID-19 (5). Recent findings have shown that higher grip strength was associated with a lower risk of COVID-19 hospitalization and a better prognosis (6, 7). Another study reported that there was no significant difference in grip strength among COVID-19 patients with different severity after 12 weeks (8). A retrospective study showed that an acute skeletal muscle loss was evident in consecutive hospitalized patients with COVID-19 compared with those without COVID-19 and contributed to poor clinical outcomes (9). COVID-19 patients with SP had a higher number of persistent symptoms than patients without SP, but that was not statistically significant (10). These observational studies show that there appears to be a correlation between COVID-19 and SP, but it is inconsistent. These results also made it elusive to assess the causal relationship between COVID‐19 and SP-related traits.

Mendelian randomization (MR) is a valid approach to infer possible causality between exposure and outcome, reducing bias from confounding factors and reverse causality in epidemiological studies (11). In the present study, we performed a two-sample MR to assess the potential causal effect between COVID‐19 and SP-related traits using instrumental variables (IVs) from the summary genome‐wide association study (GWAS) datasets. Overall, Results obtained in this study may help for identify the role of SP in the pandemic to reduce infection and attenuate clinical symptoms. It can also provide new insight into dealing with the post-acute sequelae of COVID-19 to treat or prevent the persistence of these long-lasting symptoms.

## Materials and methods

### Study design

The MR analysis was performed to explore the causality between COVID‐19 and SP-related traits. In the forward MR analysis, COVID‐19 was considered as the exposure and SP-related traits were considered as the outcome, whereas the reverse MR analysis investigated SP-related traits as the exposure and COVID‐19 as the outcome. The following 3 main assumptions were satisfied (1): the IV is tightly associated to exposure; (2) the IV is not related to any confounder of the exposure-outcome connection; (3) the IV can only affect the outcome *via* the exposure (11).

### Data source

In this MR study, GWAS summary statistics for COVID-19 phenotypes were extracted from the COVID‐19 Host Genetic Initiative (HGI) (Round 5) (12). COVID-19 phenotypes included severity (4,792-1,054,664), hospitalization (8,316-1,549,095), susceptibility (3,2494-1,316,207). The COVID-19 cases were diagnosed by laboratory confirmation or by electrical health records (using physician notes or ICD), or self-reported COVID-19 infections from the patients. Severe COVID-19 cases were defined as patients who died or required respiratory support (including bilevel positive airway pressure, continuous positive airway pressure, intubation, or high-flow nasal cannula). The controls were defined as the individuals enrolled in the cohorts and not included as cases. The COVID-19 related data was retrieved from the European population except the UK Biobank participants. The data and more information can be found online.

All the GWAS summary statistics for SP-related traits were extracted from the UK Biobank. UK Biobank is a large-scale biomedical database and research resource, globally accessible to approved researchers undertaking vital research into the most common and life-threatening diseases, containing in-depth genetic and health information from 500 000 UK participants (13). Appendicular lean mass (ALM) has been proposed as a validated and reliable indicator of muscle mass in older adults (14). ALM was quantified by appendicular fat-free mass using the bioelectrical impedance analysis with 450,243 UK Biobank individuals and adjusted by appendicular fat mass and other covariates (15). Grip strength has been widely recognized as a significant indicator of SP (16). The hand grip strength was measured with a calibrated device in a simple and non-invasive way and adjusted for hand size (17). The UK Biobank grip strength data was adjusted for age, age^2^, sex, sex × age, and sex × age^2^ (13), including 461,089 individuals of European descent for right hand grip strength and 461,026 individuals for left hand grip strength (18). The summary‐level statistics of walking pace were also obtained from the UK Biobank, including 459,915 individuals of European ancestry (18).

The genetic IVs of COVID-19 and SP-related traits were retrieved from publicly available database without sample overlapping. Ethical permission was not applicable to this study. More details for phenotype and previously ethical approval can be found in the original publications or GWAS (12, 15, 18).

### MR analysis

Independent single nucleotide polymorphisms (SNPs) at the genome‐wide significance level (p < 5 × 10^-8^) were selected as IVs for exposure (clumping r^2 =^ 0.001 and kb = 10,000) (19). Related data of IVs were also extracted from the outcome datasets without proxies. After harmonizing each pair of the exposure and outcome datasets, the inverse variance weighted (IVW) model were conducted to assess the causality. The IVW method can provide the most accurate and stable estimation of causal effects when all IVs were valid without directional pleiotropy (20). MR‐Egger intercept test was employed to evaluate horizontal pleiotropy (21). MR pleiotropy residual sum and outlier (MR-PRESSO) was conducted to identify and obtain corrected results by removing pleiotropic IVs (22). After that, The IVW method was reperformed to assess the robustness. Several sensitivity analyses were also performed, including weighted median estimation, MR‐Egger regression, MR Robust Adjusted Profile Score (RAPS). The weighted median method could control the Type 1 error rates and provide consistent causal estimates when more than 50% IVs were valid and enrolled (23). MR-Egger regression could test IVs with considerable pleiotropy and heterogeneity, whereas this approach had poor statistical power and required larger sample size (21). RAPS could tackle the idiosyncratic pleiotropy even for up to hundreds of weak IVs (24). Heterogeneity across these selected IVs was assessed by Cochran’s Q statistic. F statistic was calculated to test the strength of genetic IVs and genetic IV with F statistics > 10 was statistically considered as a strong instrument to minimize bias. The proportion of variance explained was also measured.

Actually, a small subset of SNPs were included as IVs for causal inference due to the strict inclusion and exclusion criteria, especially for COVID-19. MR Causal Analysis Using Summary Effect Estimates (CAUSE) and MR Accounting for Pleiotropy and Sample Structure simultaneously (MR-APSS) were employed to improve statistical power in the analysis, mainly by relaxing the threshold to utilize more IVs instead of only IVs at the genome‐wide significance level (25, 26). Compared with other methods, the CAUSE method could avoid more false positives and calculate the shared (non-causal) effect, accounting for correlated pleiotropy induced by confounders or unmeasured shared factors. The q value was also calculated as an estimate of the proportion of pleiotropic variants (25). Default parameters were used in the CAUSE procedures (p < 5 × 10^-3^) (https://jean997.github.io/cause/ldl_cad.html). MR-APSS was a recently proposed method in 2022, accounting for sample structure as a major confounding factor including cryptic relatedness, population stratification, and sample overlap (26). We employed the same parameters as used in an originally example recommended by the authors (p < 5 × 10^-5^) (https://github.com/YangLabHKUST/MR-APSS/blob/master/MRAPSS_Rpackage_Tutorial.pdf).

Concerning multiple testing of COVID‐19 and SP-related traits, we conservatively adjusted the p-values after the Bonferroni correction (p = 0.05/12 = 4.17E-03). The MR analyses were conducted with the R packages “MungeSumstats”, “TwosampleMR”, “CAUSE”, “MRAPSS” in the R statistical software (Version 4.1.3).

## Results

### Stage 1: a bi-directional two-sample MR analysis

**Figure 1:**
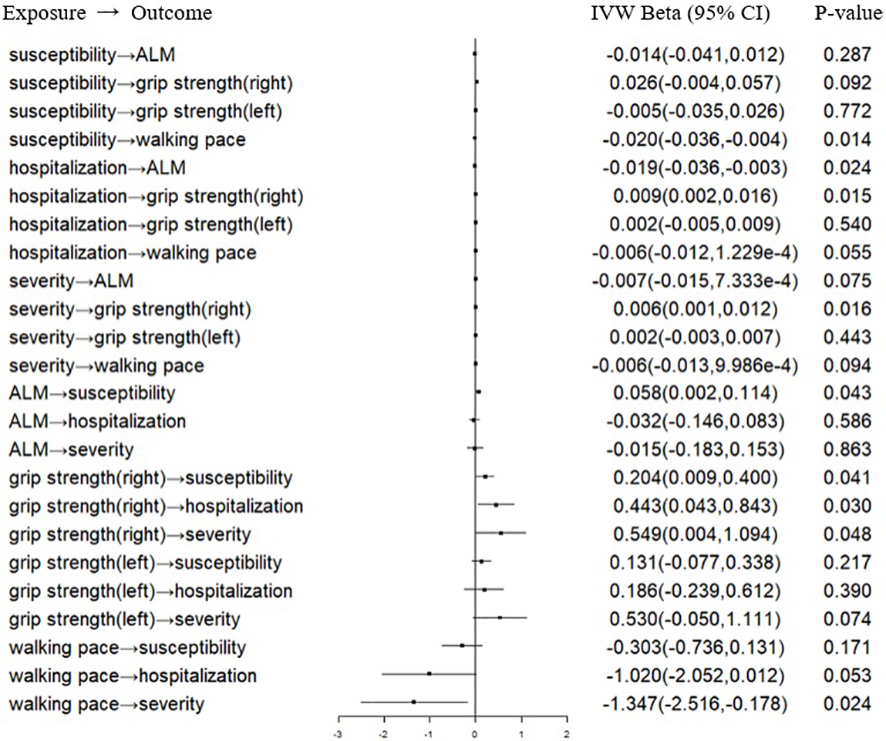
Forest plot of MR IVW analyses between COVID-19 and SP-related traits. IVW, inverse-variance weighted; CI, confidence interval; ALM, appendicular lean mass; COVID-19, coronavirus disease 2019. MR, mendelian randomization; SP, sarcopenia.

In the forward MR analysis, we analyzed the causal effect of COVID-19 on SP-related traits. The IVW results suggested that susceptibility, hospitalization and severity of COVID-19 had no causal effect on ALM, right hand grip strength, left hand grip strength and walking pace after the Bonferroni correction (**Table 1**; **Figure 1**). Consistently, the weighted median, the RAPS, the MR‐Egger, the CAUSE, the MR-APSS methods further strengthened the hypothesis that COVID-19 was not a causal risk factor for SP-related traits (**Table 2**). Notably, several results indicated the nominal causality of COVID-19 on SP-related traits using the IVW and the RAPS methods, contradicting the results with the CAUSE, the MR-APSS methods. The median shared effect ranged from –0.04 to 0.03 in the CAUSE methods, suggesting rarely bias induced by horizontal pleiotropy. The low q indicated poor correlation between genetic effects of COVID-19 on SP-related traits.

**Table 1:** Primary mendelian randomization estimates of COVID-19 on sarcopenia-related traits.

Exposures	Outcomes	IVW		Weighted median		MR‐Egger		RAPS	
		Beta (95% CI)	P	Beta (95% CI)	P	Beta (95% CI)	P	Beta (95% CI)	P
susceptibility	ALM	-0.014(-0.041, 0.012)	0.287	-0.013(-0.043, 0.017)	0.381	-0.010(-0.079, 0.059)	0.821	-0.014(-0.043, 0.014)	0.314
susceptibility	grip strength(right)	0.026(-0.004, 0.057)	0.092	NA	NA	NA	NA	0.026(-0.007, 0.060)	0.123
susceptibility	grip strength(left)	-0.005(-0.035, 0.026)	0.772	NA	NA	NA	NA	-0.005(-0.037, 0.028)	0.787
susceptibility	walking pace	-0.020(-0.036, -0.004)	0.014	-0.010(-0.030, 0.010)	0.311	0.006(-0.064, 0.077)	0.877	-0.016(-0.036, 0.004)	0.110
hospitalization	ALM	-0.019(-0.036, -0.003)	0.024	NA	NA	NA	NA	-0.020(-0.038, -0.002)	0.032
hospitalization	grip strength(right)	0.009(0.002, 0.016)	0.015	0.008(-3.972e-4, 0.016)	0.062	0.003(-0.013, 0.019)	0.711	0.009(0.001, 0.016)	0.021
hospitalization	grip strength(left)	0.002(-0.005, 0.009)	0.540	4.071e-4(-0.008, 0.009)	0.925	-0.001(-0.018, 0.015)	0.888	0.002(-0.005, 0.009)	0.620
hospitalization	walking pace	-0.006(-0.012, 1.229e-4)	0.055	-0.003(-0.010, 0.004)	0.400	-2.331e-4(-0.014, 0.014)	0.976	-0.006(-0.012, 8.030e-4)	0.087
severity	ALM	-0.007(-0.015, 7.333e-4)	0.075	-0.005(-0.014, 0.005)	0.350	-0.002(-0.032, 0.028)	0.919	-0.007(-0.019, 0.005)	0.269
severity	grip strength(right)	0.006(0.001, 0.012)	0.016	0.007(6.797e-4, 0.013)	0.030	9.228e-4(-0.013, 0.015)	0.899	0.007(0.001, 0.012)	0.019
severity	grip strength(left)	0.002(-0.003, 0.007)	0.443	2.161e-4(-0.006, 0.007)	0.948	-0.002(-0.015, 0.012)	0.806	0.002(-0.004, 0.007)	0.515
severity	walking pace	-0.006(-0.013, 9.986e-4)	0.094	-0.003(-0.008, 0.003)	0.373	0.005(-0.012, 0.023)	0.594	-0.004(-0.011, 0.002)	0.151

NA occurred because only two valid instruments were included in the analysis.

Bonferroni corrected significance level (0.05/12 = 0.004) was used to correct for multiple comparisons. p<0.004.

COVID-19, The coronavirus disease 2019; ALM, appendicular lean mass; MR, mendelian randomization; CI, confidence interval; IVW, inverse variance weighted; MR-RAPS, Mendelian Randomization Robust Adjusted Profile Score. NA, not applicable.

**Table 2:** Mendelian randomization estimates of COVID-19 on sarcopenia-related traits using the CAUSE and MR-APSS methods.

Exposures	Outcomes	CAUSE					MR-APSS			
		Median causal effect (95% CI)	Median q (CI)		P causal vs sharing		Beta (95% CI)		p	
susceptibility	ALM	0.03 (-0.53,0.48)	0.03 (0,0.22)		1		0.081 (-0.397,0.559)		0.740	
susceptibility	grip strength (right)	0.01 (-0.16,0.18)	0.04 (0,0.24)		0.94		-0.295 (-0.993,0.404)		0.408	
susceptibility	grip strength (left)	0.01 (-0.16,0.18)	0.04 (0,0.24)		0.99		-0.429 (-1.005,0.147)		0.144	
susceptibility	walking pace	-0.01 (-0.16,0.13)	0.04 (0,0.23)		0.98		-0.124 (-0.702,0.455)		0.675	
hospitalization	ALM	-0.04 (-0.2,0.09)	0.06 (0,0.24)		0.93		-0.069 (-0.735,0.598)		0.840	
hospitalization	grip strength (right)	0 (-0.10,0.09)	0.03 (0,0.23)		0.55		-0.098 (-0.455,0.260)		0.593	
hospitalization	grip strength (left)	0 (-0.10,0.10)	0.03 (0,0.22)		1.00		-0.257 (-0.592,0.077)		0.132	
hospitalization	walking pace	0 (-0.06,0.05)	0.05 (0,0.25)		0.80		-0.045 (-0.306,0.216)		0.735	
severity	ALM	0.02 (-0.15,0.07)	0.07 (0,0.26)		0.21		-0.011 (-0.465,0.443)		0.962	
severity	grip strength (right)	-0.02 (-0.04,0.02)	0.08 (0.01,0.25)		0.37		-0.023 (-0.274,0.228)		0.858	
severity	grip strength (left)	-0.02 (-0.05,0.03)	0.07 (0.01,0.25)		0.98		-0.150 (-0.386,0.097)		0.215	
severity	walking pace	-0.01 (-0.04,0.04)	0.05 (0,0.25)		0.47		-0.118 (-0.269,0.034)		0.127	

Sharing model better fit for the data in the CAUSE method.

Bonferroni corrected significance level (0.05/12 = 0.004) was used to correct for multiple comparisons. p<0.004.

COVID-19, The coronavirus disease 2019; ALM, appendicular lean mass; MR, mendelian randomization; CI, confidence interval; CAUSE, Causal Analysis Using Summary Effect Estimates; MR-APSS, MR Accounting for Pleiotropy and Sample Structure simultaneously.

In the reverse MR analysis, similar results were identified in the five MR tests, reflecting that SP-related traits had no causal effect on COVID-19 after the Bonferroni correction (**Tables 3**, **4**). The median shared effect ranged from –3.02 to 0.36, meaning bias induced by pleiotropic variants.

**Table 3:** Primary mendelian randomization estimates of sarcopenia-related traits on COVID-19.

Exposures	Outcomes	IVW		Weighted median		MR‐Egger		RAPS	
		Beta (95% CI)	P	Beta (95% CI)	P	Beta (95% CI)	P	Beta (95% CI)	P
ALM	susceptibility	0.058 (0.002, 0.114)	0.043	0.082 (-0.012, 0.176)	0.086	0.053 (-0.079, 0.186)	0.430	0.070 (0.009, 0.131)	0.024
ALM	hospitalization	-0.032 (-0.146, 0.083)	0.586	-0.080 (-0.268, 0.107)	0.402	-0.262 (-0.540, 0.016)	0.065	-0.017 (-0.146, 0.112)	0.800
ALM	severity	-0.015 (-0.183, 0.153)	0.863	-0.009 (-0.255, 0.238)	0.945	-0.299 (-0.692, 0.095)	0.138	-0.022 (-0.200, 0.157)	0.814
grip strength (right)	susceptibility	0.204 (0.009, 0.400)	0.041	0.124 (-0.172, 0.420)	0.411	0.342 (-0.406, 1.091)	0.372	0.142 (-0.070, 0.354)	0.191
grip strength (right)	hospitalization	0.443 (0.043, 0.843)	0.030	0.538 (-0.047, 1.123)	0.072	0.276 (-1.272, 1.824)	0.728	0.261 (-0.180, 0.701)	0.246
grip strength (right)	severity	0.549 (0.004, 1.094)	0.048	0.594 (-0.212, 1.400)	0.148	1.347 (-0.678, 3.373)	0.195	0.604 (0.035, 1.173)	0.038
grip strength (left)	susceptibility	0.131 (-0.077, 0.338)	0.217	0.024 (-0.286, 0.334)	0.880	0.288 (-0.586, 1.162)	0.520	0.060 (-0.187, 0.308)	0.632
grip strength (left)	hospitalization	0.186 (-0.239, 0.612)	0.390	0.378 (-0.284, 1.040)	0.263	0.067 (-1.768, 1.903)	0.943	0.186 (-0.282, 0.654)	0.436
grip strength (left)	severity	0.530 (-0.050, 1.111)	0.074	0.570 (-0.291, 1.432)	0.194	1.232 (-1.025, 3.489)	0.287	0.525 (-0.082, 1.133)	0.090
walking pace	susceptibility	-0.303 (-0.736, 0.131)	0.171	-0.238 (-0.881, 0.405)	0.469	1.441 (-0.751, 3.634)	0.205	-0.239 (-0.733, 0.256)	0.344
walking pace	hospitalization	-1.020 (-2.052, 0.012)	0.053	-1.022 (-2.273, 0.229)	0.109	4.373 (-0.413, 9.160)	0.081	-0.988 (-2.003, 0.026)	0.056
walking pace	severity	-1.347 (-2.516, -0.178)	0.024	-1.222 (-2.967, 0.524)	0.170	1.114 (-4.976, 7.204)	0.722	-1.405 (-2.690, -0.119)	0.032

Bonferroni corrected significance level (0.05/12 = 0.004) was used to correct for multiple comparisons. p<0.004.

COVID-19, The coronavirus disease 2019; ALM, appendicular lean mass; MR, mendelian randomization; CI, confidence interval; IVW, inverse variance weighted; MR-RAPS, Mendelian Randomization Robust Adjusted Profile Score.

**Table 4:** Mendelian randomization estimates of sarcopenia-related traits on COVID-19 using the CAUSE and MR-APSS methods.

Exposures	Outcomes	CAUSE					MR-APSS			
		Median causal effect (95% CI)	Median q (CI)		P causal vs sharing		Beta (95% CI)		p	
ALM	susceptibility	0.26 (-0.25,0.94)	0.08 (0,0.28)		0.28		0.008 (0.002,0.015)		0.008	
grip strength (right)	susceptibility	-0.7 (-6.8,1.99)	0.01 (0,0.18)		1		0.001 (-0.007,0.008)		0.836	
grip strength (left)	susceptibility	-1.08 (-6.53,3.57)	0.01 (0,0.16)		0.97		-0.004 (-0.015,0.008)		0.506	
walking pace	susceptibility	0.36 (-1.77,2.63)	0.04 (0,0.24)		0.57		0.019 (-0.005,0.043)		0.120	
ALM	hospitalization	-0.42 (-7.28,4.85)	0.03 (0,0.2)		1		0.013 (-0.015,0.041)		0.348	
grip strength (right)	hospitalization	-0.62 (-10.87,7.35)	0.02 (0,0.2)		1		0.016 (-0.021,0.052)		0.394	
grip strength (left)	hospitalization	0.16 (-2.28,2.46)	0.03 (0,0.22)		0.90		0.019 (-0.008,0.046)		0.176	
walking pace	hospitalization	-1.25 (-7.62,3.68)	0.03 (0,0.22)		0.75		-0.002 (-0.031,0.026)		0.887	
ALM	severity	-1.18 (-11.66,6.27)	0.02 (0,0.2)		1		0.016 (-0.023,0.056)		0.416	
grip strength (right)	severity	-0.4 (-4.38,3.2)	0.03 (0,0.22)		0.81		-0.037 (-0.089,0.016)		0.173	
grip strength (left)	severity	-2.32 (-8.05,2.36)	0.08 (0,0.32)		0.25		-0.033 (-0.102,0.035)		0.343	
walking pace	severity	-3.02 (-11.97,2.81)	0.09 (0,0.33)		0.18		-0.055 (-0.161,0.051)		0.307	

Sharing model better fit for the data in the CAUSE method.

Bonferroni corrected significance level (0.05/12 = 0.004) was used to correct for multiple comparisons. p<0.004.

COVID-19, The coronavirus disease 2019; ALM, appendicular lean mass; MR, mendelian randomization; CI, confidence interval; CAUSE, Causal Analysis Using Summary Effect Estimates; MR-APSS, MR Accounting for Pleiotropy and Sample Structure simultaneously.

### Stage 2: sensitivity analysis

To evaluate the robustness of the above results, extensive sensitivity analyses were performed, including Cochran’s Q test, MR-Egger intercept test, MR-PRESSO global test, and F statistics (**Table 5**). The Cochran’s Q test identified 2 pair of the exposure and outcome datasets so that a random-effects model was applied for them. After removing outliers detected by MR‐PRESSO (**Supplementary Table 1**), we only observed significantly horizontal pleiotropy of walking pace on hospitalization in MR-Egger intercept test. All the F statistics of selected IVs were above than 10, indicating that they were valid enough to minimize potential bias. The proportion of variance explained grew with the increasing IVs, especially in the MR-APSS, the CAUSE methods (**Supplementary Table 2**). Details of IVs were provided in Additional Tables.

**Table 5:** Sensitivity analysis of the primary causal association between COVID-19 and sarcopenia-related traits .

Exposures	Outcomes	Cochran's Q (P)	MR-Egger (P)	MR-PRESSO (P)	F
susceptibility	ALM	0.975	0.916	0.025	58.413
susceptibility	grip strength (right)	0.780	NA	<0.001	33.786
susceptibility	grip strength (left)	0.683	NA	0.019	33.786
susceptibility	walking pace	0.073	0.511	0.181	51.485
hospitalization	ALM	0.185	NA	<0.001	54.200
hospitalization	grip strength (right)	0.933	0.523	<0.001	88.914
hospitalization	grip strength (left)	0.480	0.680	<0.001	88.914
hospitalization	walking pace	0.476	0.444	0.022	87.276
severity	ALM	0.052	0.705	<0.001	68.426
severity	grip strength (right)	0.858	0.431	0.025	67.269
severity	grip strength (left)	0.697	0.576	0.040	67.269
severity	walking pace	0.028	0.247	0.007	69.587
ALM	susceptibility	0.123	0.945	0.095	101.024
ALM	hospitalization	0.064	0.073	0.022	101.717
ALM	severity	0.006	0.119	0.002	99.158
grip strength (right)	susceptibility	0.141	0.707	<0.001	47.652
grip strength (right)	hospitalization	0.141	0.826	0.019	47.716
grip strength (right)	severity	0.318	0.423	0.256	47.600
grip strength (left)	susceptibility	0.061	0.715	0.041	48.385
grip strength (left)	hospitalization	0.054	0.896	0.073	48.466
grip strength (left)	severity	0.752	0.530	0.764	48.466
walking pace	susceptibility	0.178	0.118	0.195	40.294
walking pace	hospitalization	0.038	0.030	0.039	40.294
walking pace	severity	0.289	0.423	0.321	40.294

NA occurred because only two valid instruments were included in the analysis.

COVID-19, The coronavirus disease 2019; ALM, appendicular lean mass; MR, mendelian randomization; CI, confidence interval; IVW, inverse variance weighted; MR-RAPS, Mendelian Randomization Robust Adjusted Profile Score. NA, not applicable.

## Discussion

Based on the MR results in our analysis, we conservatively summarized that there was insufficient evidence to determine a causal link between COVID-19 and SP-related traits after the Bonferroni correction. Most MR results were also nominally consistent with the conclusion. We performed the first bi-directional two-sample MR analysis to evaluate causal relationship between COVID-19 and SP-related traits, using the MR-APSS method.

Actually, several studies with related themes were reported. Three MR studies indicated that genetic evidence did not support a significant causal effect between COVID‐19 and telomere length (27–29), although the cohort study in UK Biobank showed that shorter telomere length was associated with higher risk of adverse COVID-19 outcomes (29). A MR study including 261,000 older participants estimated that telomere length would not affect grip strength, sarcopenia, or falls (30). Telomere length did not occupy a unique position in the causal relationship between COVID-19 and SP-related traits. Another three MR studies suggested that physical activity had no causal effect on COVID-19 outcomes after the Bonferroni correction, but the results nominally contradicted each other (31–33). Meanwhile, the observational study also reported a protective effect of objectively measured physical activity on COVID-19 outcomes (33). A meta-analysis of 7 randomized controlled trials showed that exercise could improve muscle mass, muscle strength, and walking speed in 3 months (34). A recent study including 435,504 UK Biobank participants observed a paradoxical result that lean mass index was not associated with COVID-19 phenotypes in a prospective cohort study while lean mass had a significant positive causal effect on COVID-19 outcomes in related MR analysis, which might require more robust MR Methods and better lean mass related data sources (35). Combined, due to ethical and practical constraints, cross-sectional designs and most low-quality randomized controlled trials could only provide correlation rather than causality. Meanwhile, although MR study could evaluate causality, it was also affected by quality of data sources and MR methods. Our MR results so far did not conflict with most findings of related MR studies or RCTs.

Undoubtedly, there were many clinical studies with high quality observed that COVID-19 was associated with acute SP in hospital and SP in long COVID-19 syndrome (9, 10). It was also observed that the presence of SP in the general population was positively correlated with the infection rate of COVID-19 (36), which contributed to poor clinical outcomes. Obviously, they appeared to form a dangerous vicious cycle. However, our MR results did not provide sufficient evidence to support a direct interaction between COVID-19 and SP, so that other factors may participate in this cycle to assist its formation. Malnutrition, reduced activity, distress and anxiety were likely to play indispensable roles in the cycle. Compared to discharged patients, patients with COVID-19 who died had higher nutritional and SP risk, lower albumin and total protein (37, 38). The muscle would atrophy significantly within two days after fixation and progress over the next 5 days (39). Patients with COVID-19 usually stay in hospital for more than 10 days (40, 41). During the acute period of COVID-19, common symptoms included depression mood (32.6%), anxiety (35.7%), insomnia (41.9%) (42). During the lockdown period, malnutrition, reduced activity, distress and anxiety were also prevalent (43–45), which was also positively correlated with susceptibility, hospitalization and severity of COVID-19 (46). These factors might act as competitive confounders in an observational study or mediating factors in a MR study. Cognitive impairment, frailty and other aging-associated diseases had the potential to serve as candidate factors (47, 48). On the basis of these observations and our MR results, we highlighted that older people should pay more attention to prevention, diagnosis, and treatment of SP instead of specific interactions between COVID-19 and SP, which would make it easier to break the dangerous vicious cycle and improve quality of life. In addition, targeting other mediating factors might also play a role. Nutritional supplementation and muscle training could provide significant improvement in muscle function and strength for COVID-19 survivors (49, 50).

In the COVID-19 pandemic, it has been reported that patients with more severe COVID-19 infection had a higher elevated serum creatine kinase level and more prone to rhabdomyolysis (51, 52), indicating that COVID-19 could cause damage to skeletal muscle. The mechanisms of individual organ damage might involve a systemic inflammatory response (53). In autopsy analysis, severe acute respiratory syndrome coronavirus 2(SARS-CoV2) virus particles were detected in organs including the heart, liver and kidney (54–56), while SARS-CoV virus particle could not be detected in skeletal muscle in SARS patients (57). Combined our MR results, we prefer that skeletal muscle injury is attributed to systemic inflammation instead of direct virus invasion. Nowadays, most countries and regions stopped requiring SARS-CoV-2 testing in public places, and mandatory quarantine (58). After vaccination, older people can adopt healthy lifestyles by increasing physical activity, improving glycemic control and body weight to reverse the situation in preparation for the next COVID-wave. Obviously, future studies across RCT or basic research from a variety of ethnic backgrounds are required to completely disentangle pathogenic mechanisms and develop effective interventions for attenuate clinical symptoms.

This study is the first bi-directional MR study to evaluate causal relationship between COVID-19 and SP-related traits, using the MR-APSS method. All parameters were set according to recommendations and thresholds were not relaxed. The COVID-19 related data was retrieved from the European population except the UK Biobank participants and the sarcopenia related data was extracted from the UK Biobank, which avoided sample overlapping and reduced bias. Extremely strict parameters and F statistic were used to ensure the validity of IV. CAUSE and MR-APSS were performed to include more IVs to improve statistical power, and consistent results were obtained. Nevertheless, there were several potential limitations in our MR analysis. First, all the data came from the European population therefore our result might only apply to Europeans. Second, it was difficult to completely remove mediation and pleiotropy so that we cannot rule out the possibility that mediating factors mediating the causality between COVID-19 and SP-related traits. Third, though the GWAS data was constantly being updated, better data sources were still required, especially for COVID-19.

## Conclusion

The mechanisms between COVID-19 and SP have not yet been fully elucidated. Our MR results did not support a direct causal relationship after the Bonferroni correction, indicating that they may indirectly interact with each other through systemic inflammatory response and other diseases. Our new insights might inform better practices to recognize, evaluate and both prevent and treat SP in the COVID-19 pandemic. We highlighted that older people should absorb adequate nutrition and strengthen exercise to cope with SP and break the dangerous vicious cycle.

## Data availability statement

The original contributions presented in the study are included in the article/**Supplementary Material**. Further inquiries can be directed to the corresponding authors.

## Author contributions

CL had the idea and drafted the final manuscript, NL performed data analysis, YZ and BX created the tables. Other authors gave constructive suggestions during the process. TX and HL drafted the final manuscript. All authors contributed to the article and approved the submitted version.

## References

[1] WHO coronavirus COVID-19 dashboard 2022 . Available at: https://covid19.who.int/.

[2] Mak JKL Kuja-Halkola R Wang YZ Hagg S Jylhava J. Frailty and comorbidity in predicting community COVID-19 mortality in the UK biobank: the effect of sampling. J Am Geriatr Soc (2021) 69(5):1128–39. doi: 10.1111/jgs.17089 PMC8013405 33619733

[3] Han Q Zheng B Daines L Sheikh A. Long-term sequelae of COVID-19: a systematic review and meta-analysis of one-year follow-up studies on post-COVID symptoms. Pathogens (2022) 11(2):269. doi: 10.3390/pathogens11020269 35215212 PMC8875269

[4] Fernández-Lázaro D Garrosa E Seco-Calvo J Garrosa M. Potential satellite cell-linked biomarkers in aging skeletal muscle tissue: proteomics and proteogenomics to monitor sarcopenia. Proteomes (2022) 10(3):29. doi: 10.3390/proteomes10030029 35997441 PMC9396989

[5] Soares MN Eggelbusch M Naddaf E Gerrits KHL van der Schaaf M van den Borst B. Skeletal muscle alterations in patients with acute covid-19 and post-acute sequelae of covid-19. J Cachexia Sarcopenia Muscle (2022) 13(1):11–22. doi: 10.1002/jcsm.12896 34997689 PMC8818659

[6] Pucci G D’Abbondanza M Curcio R Alcidi R Campanella T Chiatti L. Handgrip strength is associated with adverse outcomes in patients hospitalized for COVID-19-associated pneumonia. Intern Emerg Med (2022) 17(7):1997–2004. doi: 10.1007/s11739-022-03060-3 PMC9362345 35930184

[7] Cheval B Sieber S Maltagliati S Millet GP Formanek T Chalabaev A. Muscle strength is associated with COVID-19 hospitalization in adults 50 years of age or older. J Cachexia Sarcopenia Muscle (2021) 12(5):1136–43. doi: 10.1002/jcsm.12738 PMC8426913 34363345

[8] Karasu AU Karataş L Yildiz Y Günendi Z. Natural course of muscular strength, physical performance, and musculoskeletal symptoms in hospitalized patients with COVID-19 Arch phys med rehabil. (2022) 104(1):18–26. doi: 10.1016/j.apmr.2022.09.001 PMC9464470 36103903

[9] Attaway A Welch N Dasarathy D Amaya-Hughley J Bellar A Biehl M. Acute skeletal muscle loss in SARS-CoV-2 infection contributes to poor clinical outcomes in COVID-19 patients. J Cachexia Sarcopenia Muscle (2022) 13(5):2436–46. doi: 10.1002/jcsm.13052 PMC9350025 35851995

[10] Martone AM Tosato M Ciciarello F Galluzzo V Zazzara MB Pais C. Sarcopenia as potential biological substrate of long COVID-19 syndrome: prevalence, clinical features, and risk factors. J Cachexia Sarcopenia Muscle (2022) 13(4):1974–82. doi: 10.1002/jcsm.12931 PMC9349974 35698920

[11] Skrivankova VW Richmond RC Woolf BAR Yarmolinsky J Davies NM Swanson SA. Strengthening the reporting of observational studies in epidemiology using mendelian randomization: the STROBE-MR statement. JAMA (2021) 326(16):1614–21. doi: 10.1001/jama.2021.18236 34698778

[12] Initiative C-HG. Mapping the human genetic architecture of COVID-19. Nature (2021) 600(7889):472–7. doi: 10.1038/s41586-021-03767-x PMC8674144 34237774

[13] Sudlow C Gallacher J Allen N Beral V Burton P Danesh J. UK Biobank: an open access resource for identifying the causes of a wide range of complex diseases of middle and old age. PloS Med (2015) 12(3):e1001779. doi: 10.1371/journal.pmed.1001779 25826379 PMC4380465

[14] Cawthon PM Peters KW Shardell MD McLean RR Dam TT Kenny AM. Cutpoints for low appendicular lean mass that identify older adults with clinically significant weakness. J Gerontol A Biol Sci Med Sci (2014) 69(5):567–75. doi: 10.1093/gerona/glu023 PMC3991141 24737559

[15] Pei YF Liu YZ Yang XL Zhang H Feng GJ Wei XT. The genetic architecture of appendicular lean mass characterized by association analysis in the UK biobank study. Commun Biol (2020) 3(1):608. doi: 10.1038/s42003-020-01334-0 33097823 PMC7585446

[16] Cruz-Jentoft AJ Bahat G Bauer J Boirie Y Bruyere O Cederholm T. Sarcopenia: revised European consensus on definition and diagnosis. Age Ageing (2019) 48(1):16–31. doi: 10.1093/ageing/afy169 30312372 PMC6322506

[17] Biobank U. Grip strength measurement [updated 2011 . Available at: https://biobank.ndph.ox.ac.uk/showcase/ukb/docs/Gripstrength.pdf.

[18] Mitchell RE EB Mitchell R Raistrick CA Paternoster L Hemani G Gaunt TR. MRC IEU UK biobank GWAS pipeline version 2. University of Bristol (2019). doi: 10.5523/bris.pnoat8cxo0u52p6ynfaekeigi

[19] Clarke L Zheng-Bradley X Smith R Kulesha E Xiao C Toneva I. The 1000 genomes project: data management and community access. Nat Methods (2012) 9(5):459–62. doi: 10.1038/nmeth.1974 PMC3340611 22543379

[20] Burgess S Butterworth A Thompson SG. Mendelian randomization analysis with multiple genetic variants using summarized data. Genet Epidemiol (2013) 37(7):658–65. doi: 10.1002/gepi.21758 PMC4377079 24114802

[21] Bowden J Davey Smith G Burgess S. Mendelian randomization with invalid instruments: effect estimation and bias detection through egger regression. Int J Epidemiol (2015) 44(2):512–25. doi: 10.1093/ije/dyv080 PMC4469799 26050253

[22] Verbanck M Chen CY Neale B Do R. Detection of widespread horizontal pleiotropy in causal relationships inferred from mendelian randomization between complex traits and diseases. Nat Genet (2018) 50(5):693–8. doi: 10.1038/s41588-018-0099-7 PMC6083837 29686387

[23] Bowden J Davey Smith G Haycock PC Burgess S. Consistent estimation in mendelian randomization with some invalid instruments using a weighted median estimator. Genet Epidemiol (2016) 40(4):304–14. doi: 10.1002/gepi.21965 PMC4849733 27061298

[24] Zhao Q Wang J Hemani G Bowden J Small DS. Statistical inference in two-sample summary-data mendelian randomization using robust adjusted profile score. Ann Statistics (2020) 48(3):1742–69. doi: 10.1214/19-AOS1866

[25] Morrison J Knoblauch N Marcus JH Stephens M He X. Mendelian randomization accounting for correlated and uncorrelated pleiotropic effects using genome-wide summary statistics. Nat Genet (2020) 52(7):740–7. doi: 10.1038/s41588-020-0631-4 PMC7343608 32451458

[26] Hu X Zhao J Lin Z Wang Y Peng H Zhao H. Mendelian randomization for causal inference accounting for pleiotropy and sample structure using genome-wide summary statistics. Proc Natl Acad Sci U S A (2022) 119(28):e2106858119. doi: 10.1073/pnas.2106858119 35787050 PMC9282238

[27] Huang D Lin S He J Wang Q Zhan Y. Association between COVID-19 and telomere length: a bidirectional mendelian randomization study. J Med Virol (2022) 94(11):5345–53. doi: 10.1002/jmv.28008 PMC9349767 35854470

[28] Jiang L Tang BS Guo JF Li JC. Telomere length and COVID-19 outcomes: a two-sample bidirectional mendelian randomization study. Front Genet (2022) 13:805903. doi: 10.3389/fgene.2022.805903 35677559 PMC9168682

[29] Wang Q Codd V Raisi-Estabragh Z Musicha C Bountziouka V Kaptoge S. Shorter leukocyte telomere length is associated with adverse COVID-19 outcomes: a cohort study in UK biobank. EBioMedicine (2021) 70:103485. doi: 10.1016/j.ebiom.2021.103485 34304048 PMC8299112

[30] Kuo CL Pilling LC Kuchel GA Ferrucci L Melzer D. Telomere length and aging-related outcomes in humans: a mendelian randomization study in 261,000 older participants. Aging Cell (2019) 18(6):e13017. doi: 10.1111/acel.13017 31444995 PMC6826144

[31] Li S Hua X. Modifiable lifestyle factors and severe COVID-19 risk: a mendelian randomisation study. BMC Med Genomics (2021) 14(1):38. doi: 10.1186/s12920-021-00887-1 33536004 PMC7856619

[32] Chen X Hong X Gao W Luo S Cai J Liu G. Causal relationship between physical activity, leisure sedentary behaviors and COVID-19 risk: a mendelian randomization study. J Transl Med (2022) 20(1):216. doi: 10.1186/s12967-022-03407-6 35562752 PMC9100292

[33] Zhang X Li X Sun Z He Y Xu W Campbell H. Physical activity and COVID-19: an observational and mendelian randomisation study. J Glob Health (2020) 10(2):020514. doi: 10.7189/jogh.10.020514 33312507 PMC7719276

[34] Yoshimura Y Wakabayashi H Yamada M Kim H Harada A Arai H. Interventions for treating sarcopenia: a systematic review and meta-analysis of randomized controlled studies. J Am Med Dir Assoc (2017) 18(6):553 e1– e16. doi: 10.1016/j.jamda.2017.03.019 28549707

[35] Gao M Wang Q Piernas C Astbury NM Jebb SA Holmes MV. Associations between body composition, fat distribution and metabolic consequences of excess adiposity with severe COVID-19 outcomes: observational study and mendelian randomisation analysis. Int J Obes (Lond) (2022) 46(5):943–50. doi: 10.1038/s41366-021-01054-3 PMC8758930 35031696

[36] Xu Y Xu JW You P Wang BL Liu C Chien CW. Prevalence of sarcopenia in patients with COVID-19: a systematic review and meta-analysis. Front Nutr (2022) 9:925606. doi: 10.3389/fnut.2022.925606 35859753 PMC9289534

[37] da Silva CL Sousa TMM de Sousa Junior JB Nakano EY. Nutritional factors associated with mortality in hospitalized patients with COVID-19. Clin Nutr Open Sci (2022) 45:17–26. doi: 10.1016/j.nutos.2022.08.001 36035064 PMC9391077

[38] Vong T Yanek LR Wang L Yu H Fan C Zhou E. Malnutrition increases hospital length of stay and mortality among adult inpatients with COVID-19. Nutrients (2022) 14(6):1310. doi: 10.3390/nu14061310 35334967 PMC8949069

[39] Kilroe SP Fulford J Jackman SR LJC VANL Wall BT. Temporal muscle-specific disuse atrophy during one week of leg immobilization. Med Sci Sports Exerc (2020) 52(4):944–54. doi: 10.1249/MSS.0000000000002200 31688656

[40] Zhao W Zha X Wang N Li D Li A Yu S. Clinical characteristics and durations of hospitalized patients with COVID-19 in Beijing: a retrospective cohort study. Cardiovasc Innov Applications (2021) 6(1):33–44. doi: 10.15212/CVIA.2021.0019

[41] Thai PQ Toan DTT Son DT Van HTH Minh LN Hung LX. Factors associated with the duration of hospitalisation among COVID-19 patients in Vietnam: a survival analysis. Epidemiol Infect (2020) 148:e114. doi: 10.1017/S0950268820001259 32517822 PMC7306545

[42] Rogers JP Chesney E Oliver D Pollak TA McGuire P Fusar-Poli P. Psychiatric and neuropsychiatric presentations associated with severe coronavirus infections: a systematic review and meta-analysis with comparison to the COVID-19 pandemic. Lancet Psychiatry (2020) 7(7):611–27. doi: 10.1016/S2215-0366(20)30203-0 PMC7234781 32437679

[43] Verma S Mishra A. Depression, anxiety, and stress and socio-demographic correlates among general Indian public during COVID-19. Int J Soc Psychiatry (2020) 66(8):756–62. doi: 10.1177/0020764020934508 32567466

[44] Ghanem J Colicchio B Andrès E Geny B Dieterlen A. Lockdown effect on elderly nutritional health. J Clin Med (2021) 10(21):5052. doi: 10.3390/jcm10215052 34768572 PMC8584610

[45] Kite C Lagojda L Clark CCT Uthman O Denton F McGregor G. Changes in physical activity and sedentary behaviour due to enforced COVID-19-Related lockdown and movement restrictions: a protocol for a systematic review and meta-analysis. Int J Environ Res Public Health (2021) 18(10):5251. doi: 10.3390/ijerph18105251 34069251 PMC8155982

[46] Barrea L Vetrani C Caprio M Cataldi M Ghoch ME Elce A. From the ketogenic diet to the Mediterranean diet: the potential dietary therapy in patients with obesity after CoVID-19 infection (Post CoVID syndrome). Curr Obes Rep (2022) 11(3):144–65. doi: 10.1007/s13679-022-00475-z PMC9075143 35524067

[47] Ciarambino T Crispino P Minervini G Giordano M. COVID-19 and frailty. Vaccines (Basel) (2023) 11(3):606. doi: 10.3390/vaccines11030606 36992190 PMC10057998

[48] Kashtanova DA Erema VV Gusakova MS Sutulova ER Yakovchik AY Ivanov MV. Mortality and survival in nonagenarians during the COVID-19 pandemic: unstable equilibrium of aging. Front Med (Lausanne) (2023) 10:1132476. doi: 10.3389/fmed.2023.1132476 36936206 PMC10018166

[49] Galluzzo V Zazzara MB Ciciarello F Savera G Pais C Calvani R. Fatigue in covid-19 survivors: the potential impact of a nutritional supplement on muscle strength and function. Clin Nutr ESPEN (2022) 51:215–21. doi: 10.1016/j.clnesp.2022.08.029 PMC9428328 36184207

[50] Del Corral T Garrido RF Plaza-Manzano G Fernández-de-Las-Peñas C Navarro-Santana M López-de-Uralde-Villanueva I. Home-based respiratory muscle training on quality of life and exercise tolerance in long-term post-COVID-19: randomized controlled trial. Ann Phys Rehabil Med (2023) 66(1):101709. doi: 10.1016/j.rehab.2022.101709 36191860 PMC9708524

[51] Mao L Jin H Wang M Hu Y Chen S He Q. Neurologic manifestations of hospitalized patients with coronavirus disease 2019 in wuhan, China. JAMA Neurol (2020) 77(6):683–90. doi: 10.1001/jamaneurol.2020.1127 PMC7149362 32275288

[52] Suwanwongse K Shabarek N. Rhabdomyolysis as a presentation of 2019 novel coronavirus disease. Cureus (2020) 12(4):e7561. doi: 10.7759/cureus.7561 32382463 PMC7202588

[53] Liu J Li S Liu J Liang B Wang X Wang H. Longitudinal characteristics of lymphocyte responses and cytokine profiles in the peripheral blood of SARS-CoV-2 infected patients. EBioMedicine (2020) 55:102763. doi: 10.1016/j.ebiom.2020.102763 32361250 PMC7165294

[54] Puelles VG Lütgehetmann M Lindenmeyer MT Sperhake JP Wong MN Allweiss L. Multiorgan and renal tropism of SARS-CoV-2. New Engl J Med (2020) 383(6):590–2. doi: 10.1056/NEJMc2011400 PMC7240771 32402155

[55] Farkash EA Wilson AM Jentzen JM. Ultrastructural evidence for direct renal infection with SARS-CoV-2. J Am Soc Nephrol (2020) 31(8):1683–7. doi: 10.1681/ASN.2020040432 PMC7460898 32371536

[56] Wichmann D Sperhake J-P Lütgehetmann M Steurer S Edler C Heinemann A. Autopsy findings and venous thromboembolism in patients with COVID-19. Ann Internal Med (2020) 173(4):268–77. doi: 10.7326/M20-2003 PMC7240772 32374815

[57] Ding Y He L Zhang Q Huang Z Che X Hou J. Organ distribution of severe acute respiratory syndrome (SARS) associated coronavirus (SARS-CoV) in SARS patients: implications for pathogenesis and virus transmission pathways. J Pathol (2004) 203(2):622–30. doi: 10.1002/path.1560 PMC7167761 15141376

[58] Wang G Yao Y Wang Y Gong J Meng Q Wang H. Determinants of COVID-19 vaccination status and hesitancy among older adults in China. Nat Med (2023) 29(3):623–31. doi: 10.1038/s41591-023-02241-7 PMC10285745 36720270

