# Supplementary material for: COVID‐19 and sarcopenia-related traits: a bidirectional Mendelian randomization study
Source: Front Endocrinol (Lausanne). 2023 May 10;14:1162936. doi: 10.3389/fendo.2023.1162936 (PMC10206246; doi:10.3389/fendo.2023.1162936)
Supplement: Supplementary file 2 [file Table_1.docx]

Supplementary Table 1. IVs outliers in MR-PRESSO analysis.

| Exposures | Outcomes | No. of IVs | The IVs outliers |
| --- | --- | --- | --- |
| susceptibility | ALM | 1 | rs2109069 |
| susceptibility | grip strength (right) | 2 | rs35508621, rs579459 |
| susceptibility | grip strength (left) | 2 | rs35508621, rs579459 |
| susceptibility | walking pace | 0 | NA |
| hospitalization | ALM | 5 | rs111837807, rs2109069, rs35081325, rs41264915, rs622568 |
| hospitalization | grip strength (right) | 2 | rs111837807, rs41264915 |
| hospitalization | grip strength (left) | 2 | rs111837807, rs41264915 |
| hospitalization | walking pace | 2 | rs1859330, rs41264915 |
| severity | ALM | 3 | rs111837807, rs2109069, rs622568 |
| severity | grip strength (right) | 1 | rs111837807 |
| severity | grip strength (left) | 1 | rs111837807 |
| severity | walking pace | 1 | rs77534576 |
| ALM | susceptibility | 0 | NA |
| ALM | hospitalization | 1 | rs11580040 |
| ALM | severity | 1 | rs11187838 |
| grip strength (right) | susceptibility | 1 | rs600038 |
| grip strength (right) | hospitalization | 1 | rs600038 |
| grip strength (right) | severity | 0 | NA |
| grip strength (left) | susceptibility | 0 | NA |
| grip strength (left) | hospitalization | 0 | NA |
| grip strength (left) | severity | 0 | NA |
| walking pace | susceptibility | 0 | NA |
| walking pace | hospitalization | 0 | NA |
| walking pace | severity | 0 | NA |

Abbreviations: IVs, instrumental variables; MR, mendelian randomization; ALM, appendicular lean mass; MR-PRESSO, MR pleiotropy residual sum and outlier; NA, not applicable.

Supplementary Table 2. Variance explained by IVs.

| Exposure | outcome | primary MR methods | | | CAUSE | | | MR-APSS | | |
| --- | --- | --- | --- | --- | --- | --- | --- | --- | --- | --- |
|  |  | No. of IVs | R2. exposure | R2. outcome | No. of IVs | R2. exposure | R2. outcome | No. of IVs | R2. exposure | R2. outcome |
| susceptibility | ALM | 3 | 1.398e-4 | 2.627e-6 | 1454 | 0.017 | 0.010 | 31 | 5.686e-4 | 1.249e-4 |
| susceptibility | grip strength (right) | 2 | 5.424e-5 | 6.332e-6 | 1389 | 0.016 | 0.005 | 32 | 5.838e-4 | 2.372e-4 |
| susceptibility | grip strength (left) | 2 | 5.424e-5 | 5.426e-7 | 1389 | 0.016 | 0.005 | 32 | 5.838e-4 | 2.213e-4 |
| susceptibility | walking pace | 4 | 1.646e-4 | 2.821e-5 | 1389 | 0.016 | 0.004 | 32 | 5.838e-4 | 3.058e-4 |
| hospitalization | ALM | 2 | 1.240e-4 | 1.524e-5 | 1571 | 0.026 | 0.011 | 32 | 0.001 | 6.265e-4 |
| hospitalization | grip strength (right) | 5 | 6.756e-4 | 1.456e-5 | 1545 | 0.026 | 0.005 | 33 | 0.001 | 2.607e-4 |
| hospitalization | grip strength (left) | 5 | 6.756e-4 | 8.369e-6 | 1545 | 0.026 | 0.006 | 33 | 0.001 | 2.307e-4 |
| hospitalization | walking pace | 5 | 6.662e-4 | 1.566e-5 | 1545 | 0.026 | 0.005 | 33 | 0.001 | 1.657e-4 |
| severity | ALM | 5 | 9.036e-4 | 2.793e-5 | 1637 | 0.064 | 0.011 | 42 | 0.003 | 5.870e-4 |
| severity | grip strength (right) | 7 | 0.001 | 1.819e-5 | 1591 | 0.062 | 0.005 | 42 | 0.003 | 2.355e-4 |
| severity | grip strength (left) | 7 | 0.001 | 9.618e-6 | 1591 | 0.062 | 0.005 | 42 | 0.003 | 2.727e-4 |
| severity | walking pace | 7 | 0.001 | 4.525e-5 | 1591 | 0.062 | 0.005 | 42 | 0.003 | 1.720e-4 |
| ALM | susceptibility | 462 | 0.105 | 4.087e-4 | 8045 | 0.488 | 0.007 | 1930 | 0.213 | 0.002 |
| ALM | hospitalization | 440 | 0.100 | 5.904e-4 | 7848 | 0.480 | 0.010 | 1919 | 0.212 | 0.002 |
| ALM | severity | 461 | 0.103 | 0.002 | 8170 | 0.492 | 0.025 | 1913 | 0.212 | 0.006 |
| grip strength (right) | susceptibility | 129 | 0.014 | 1.346e-4 | 3802 | 0.138 | 0.003 | 695 | 0.041 | 6.435e-4 |
| grip strength (right) | hospitalization | 128 | 0.014 | 1.925e-4 | 3734 | 0.138 | 0.005 | 693 | 0.040 | 9.567e-4 |
| grip strength (right) | severity | 130 | 0.014 | 4.085e-4 | 3861 | 0.140 | 0.011 | 693 | 0.040 | 0.002 |
| grip strength (left) | susceptibility | 114 | 0.012 | 1.221e-4 | 3814 | 0.137 | 0.003 | 699 | 0.040 | 6.578e-4 |
| grip strength (left) | hospitalization | 113 | 0.012 | 1.639e-4 | 3747 | 0.134 | 0.005 | 697 | 0.040 | 9.480e-4 |
| grip strength (left) | severity | 114 | 0.012 | 2.918e-4 | 3876 | 0.138 | 0.011 | 700 | 0.040 | 0.002 |
| walking pace | susceptibility | 42 | 0.004 | 4.230e-5 | 3098 | 0.102 | 0.003 | 472 | 0.023 | 4.321e-4 |
| walking pace | hospitalization | 42 | 0.004 | 8.151e-5 | 3050 | 0.100 | 0.004 | 468 | 0.023 | 6.480e-4 |
| walking pace | severity | 42 | 0.004 | 1.450e-4 | 3147 | 0.103 | 0.009 | 469 | 0.023 | 0.002 |

Abbreviations: IVs, instrumental variables; ALM, appendicular lean mass; MR, mendelian randomization; CAUSE, Causal Analysis Using Summary Effect Estimates; MR-APSS, MR Accounting for Pleiotropy and Sample Structure simultaneously. Vector of R values were calculated by the code in the TwosampleMR R package: *get_r_from_pn*.
